# Grape Seed Proanthocyanidins Enhance Time-Dependent HO-1 Activation and Improve Redox Homeostasis in Obesity-Induced Hepatic Dysfunction

**DOI:** 10.3390/antiox15060734

**Published:** 2026-06-09

**Authors:** María Zamora-Úbeda, Aina Gironès-Garreta, Julieta Cirasino, Josep M. Del Bas, Jorge R. Soliz-Rueda, Miquel Mulero, Enrique Calvo

**Affiliations:** 1 Nutrigenomics Research Group, Department of Biochemistry and Biotechnology, Universitat Rovira i Virgili, 43007 Tarragona, Spain; maria.zamora@urv.cat (M.Z.-Ú.); aina.gironesi@urv.cat (A.G.-G.); julieta.cirasino@urv.cat (J.C.); josepm.delbas@urv.cat (J.M.D.B.); jorgericardo.soliz@urv.cat (J.R.S.-R.); 2 Southern Catalonia Biomedical Research Institute (IRB CatSud), 43204 Tarragona, Spain; 3 Center of Environmental, Food and Toxicological Technology (TecnATox), Universitat Rovira i Virgili, 43007 Tarragona, Spain

**Keywords:** metabolic dysfunction-associated steatotic liver disease (MASLD), cafeteria diet, grape seed proanthocyanidin extract (GSPE), circadian rhythm, NRF2/HO-1 axis, oxidative stress, autophagy, endoplasmic reticulum stress, hepatic metabolomics, AMPK signaling

## Abstract

Metabolic dysfunction-associated steatotic liver disease (MASLD) is characterized by impaired metabolic flexibility, oxidative stress, and disruption of the temporal coordination of hepatic processes. Obesogenic diets contribute to this dysfunction by altering redox homeostasis and autophagy, thereby promoting lipid accumulation and cellular stress. In this study, we investigated whether grape seed proanthocyanidin extract (GSPE), a polyphenol-rich compound with antioxidant properties, can modulate these alterations in a time-dependent manner. Male Fischer 344 rats were fed a standard or cafeteria diet and supplemented with GSPE (25 mg/kg) at the onset of the active phase (ZT12). Liver samples were collected across four Zeitgeber times to evaluate circadian-related proteins, autophagy markers, antioxidant responses, lipid content, and metabolomic profiles. Cafeteria feeding disrupts hepatic homeostasis, reducing BMAL1 protein levels, altering the temporal organization of autophagy markers, and impairing redox regulation. GSPE did not restore core clock protein expression but induced a pronounced, time-specific activation of the NRF2/HO-1 axis, with a marked increase in HO-1 at the onset of the active phase. This effect was associated with a metabolic shift toward amino acid-related pathways linked to redox balance. These findings indicate that GSPE enhances antioxidant defenses in a time-dependent manner, improving redox–metabolic coordination under obesogenic conditions.

## 1. Introduction

Metabolic dysfunction-associated steatotic liver disease (MASLD) is a highly prevalent metabolic disorder characterized not only by hepatic lipid accumulation but also by profound alterations in metabolic homeostasis. Increasing evidence indicates that MASLD progression is tightly linked to disruption of temporal metabolic organization, whereby nutrient overload and obesogenic diets impair the coordination of circadian, metabolic, and cellular stress-response pathways [1,2]. The hepatic circadian clock orchestrates daily oscillations in lipid metabolism, mitochondrial function, and redox balance, and its disruption contributes to metabolic inflexibility and disease progression [3]. At the molecular level, the core clock component brain and muscle ARNT-like 1 (BMAL1) plays a pivotal role in synchronizing hepatic metabolism with feeding–fasting cycles. Disruption of BMAL1 signaling has been associated with impaired metabolic flexibility, altered lipid handling, and increased susceptibility to hepatic steatosis [4,5]. Beyond metabolic regulation, circadian clocks also coordinate cellular quality-control systems, including autophagy. Hepatic autophagy exhibits circadian rhythmicity, enabling the timely removal of damaged organelles and the recycling of metabolic substrates in accordance with nutrient availability [6]. Disruption of this temporal organization, as observed under obesogenic conditions, may compromise hepatocellular homeostasis. In this regard, autophagy, and particularly lipophagy, plays a central role in hepatic lipid metabolism by facilitating the degradation of lipid droplets and supporting mitochondrial *β*-oxidation. Impaired autophagic flux has been consistently associated with hepatic steatosis, as defective lipophagy limits triglyceride mobilization and promotes lipid accumulation [7,8]. In parallel, endoplasmic reticulum (ER) stress contributes to metabolic dysfunction through activation of the unfolded protein response (UPR), which becomes maladaptive under chronic nutrient overload and further disrupts proteostasis and lipid metabolism [9]. Closely linked to these processes, oxidative stress represents a key pathological feature connecting nutrient overload with hepatocellular damage. Excessive substrate influx into mitochondria promotes reactive oxygen species (ROS) production, lipid peroxidation, and inflammatory signaling [10]. A central regulator of the cellular antioxidant response is nuclear factor erythroid 2-related factor 2 (NRF2), which controls the expression of cytoprotective genes, including heme oxygenase-1 (HO-1), glutathione-related enzymes, and detoxification pathways [11]. Importantly, NRF2 signaling is functionally interconnected with autophagy through the p62–KEAP1 axis, whereby p62 accumulation promotes NRF2 stabilization and activation, linking proteostasis with antioxidant defenses [12,13]. Emerging evidence further indicates that NRF2 activity is under circadian control. Core clock components such as BMAL1 regulate NRF2-dependent transcription, while NRF2-driven antioxidant responses exhibit time-of-day-dependent oscillations, establishing a bidirectional relationship between circadian timing and redox homeostasis [14,15,16]. Interestingly, circadian misalignment has been shown to impair antioxidant defenses and exacerbate metabolic liver disease, highlighting the importance of temporally coordinated stress responses [17]. In this context, natural bioactive compounds have gained increasing attention as modulators of metabolic and redox homeostasis. Polyphenols, including proanthocyanidins, exert antioxidant, anti-inflammatory, and metabolic regulatory effects through modulation of NRF2 signaling, autophagy, and mitochondrial function [18,19,20]. Grape seed proanthocyanidin extract (GSPE), in particular, has demonstrated hepatoprotective effects, including attenuation of oxidative stress and improvement of lipid metabolism [21]. Importantly, recent studies indicate that GSPE can modulate hepatic circadian regulation and antioxidant responses, suggesting a chrono-modulatory role under metabolic stress conditions [22,23,24]. Despite these advances, how obesogenic diets disrupt the temporal coordination of hepatic circadian, autophagic, and redox pathways, and whether GSPE can restore their integrated regulation, remains incompletely understood. In particular, the temporal dynamics of antioxidant responses, including NRF2 activation and HO-1 expression, have been poorly explored in MASLD. Therefore, in the present study, we investigated the impact of a cafeteria diet (CAF) on the temporal regulation of hepatic circadian signaling, autophagy, redox homeostasis, and metabolism in Fischer 344 rats, and evaluated whether GSPE supplementation can restore the coordinated rhythmicity of these pathways. Special emphasis was placed on the NRF2/HO-1 axis as a potential mechanistic link between circadian regulation and antioxidant defense, providing insight into time-dependent adaptive responses in the liver under obesogenic conditions.

## 2. Materials and Methods

### 2.1. Experimental Procedure

Forty-eight 12-week-old male Fischer 344 rats (Janvier, Barcelona, Spain) were placed in pairs at 23 °C, 55% humidity, under a standard photoperiod of 12 h of light and 12 h of darkness. After arrival, the rats went through a one-week adaptation period with *ad libitum* food and drink access, after which they were randomly assigned to two groups depending on the diet. Thus, 16 rats were fed a standard (STD) diet, whose composition was 20% protein, 8% fat and 72% carbohydrates (Panlab, Barcelona, Spain), while 32 rats were fed a cafeteria (CAF) diet composed of 11% protein, 31% fat and 58% carbohydrates. This CAF diet included various foods commonly consumed by humans: biscuits with cheese and pâte, bacon, ensaimada (coiled puff pastry) (Hacendado, Valencia, Spain)), standard chow, carrots, and milk with a concentration of 22% sugar *w*/*v*. These diets were given for 5 weeks. After the fifth week and for 4 weeks, the 16 STD-fed rats received a vehicle (VH) treatment consisting of condensed milk vehicle diluted in water (1:5 *v*/*v*); 16 rats in the CAF group received a treatment with 25 mg/kg GSPE (Les Dérivés Résiniques et Terpéniques, Dax, France) diluted 1/5 in condensed milk, while the other 16 rats received the VH treatment. The GSPE phenolic profile was characterized by HPLC-ESI-MS/MS (Agilent Technologies, Palo Alto, CA, USA), as detailed in Supplementary Note S1, following the methodology previously described by Margalef et al. [25]. Furthermore, these treatments were given at the beginning of the active phase (8 p.m. or ZT12) through oral administration with a syringe (Comercial Bellés, Tarragona, Spain). During the whole experiment, body weight and food intake were registered weekly. Finally, after their last treatment dose, the rats were fasted for 3 h and sacrificed by decapitation at different time points (9 a.m. or ZT1, 3 p.m. or ZT7, 9 p.m. or ZT13, and 3 a.m. or ZT19), thus dividing each group—STD-VH, CAF-VH and CAF-GSPE—into 4 additional sub-groups of four rats each, according to the time they were sacrificed. Livers were kept at −80 °C for further research. All the animal care and experimental protocols were authorized by the Ethics Review Committee for Animal Experimentation of the Universitat Rovira i Virgili (reference number 9495, 18 September 2019) and handled according to Directive 2010/63/EU [26] of the Council of the European Union and the procedure established by the Department d’Agricultura, Ramaderia i Pesca of the Generalitat de Catalunya.

### 2.2. Hepatic Nuclear Isolation

For each liver sample, 90 mg of tissue was homogenized in sucrose homogenization buffer containing 0.25 M sucrose, 25 mM KCl, 10 mM HEPES, and 5 mM MgCl_2_ (pH 7.4). To prevent protein degradation and dephosphorylation, the buffer was supplemented with 1 mM phenylmethanesulfonyl fluoride (PMSF), 1% (*v*/*v*) protease inhibitor cocktail (PIC), and 1% (*v*/*v*) each of phosphatase inhibitor cocktails 2 and 3 and (all obtained from Sigma-Aldrich, Madrid, Spain). Homogenates were centrifuged at 1000× *g* for 10 min at 4 °C to obtain the nuclear pellets. The pellets were then resuspended in RIPA buffer (50 mM Tris-HCl, 150 mM NaCl, pH 7.4, 1% Tween 20, 0.25% Na-deoxycholate), composed of phenylmethanesulfonyl fluoride (PMSF), phosphatase inhibitor cocktails 2 and 3, and protease inhibitor cocktail (PIC), all obtained from Sigma-Aldrich (Madrid, Spain). Protein concentration was determined using a bicinchoninic acid (BCA) protein assay kit (Thermo Fisher Scientific, Madrid, Spain).

### 2.3. Total Tissue Lysate Protein Extraction

For each liver sample, 60 mg of tissue was homogenized in RIPA buffer with TissueLyser LT (QIAGEN, Madrid, Spain). Lysates were centrifuged at 20,000× *g* for 15 min at 4 °C, and the supernatants were collected.

### 2.4. Protein Quantification and Western Blot Analysis

Protein concentrations from both total tissue lysates and isolated nuclear fractions were determined using a bicinchoninic acid (BCA) protein assay kit (Thermo Fisher Scientific, Madrid, Spain). Equal amounts of total proteins, 50 μg for total lysate and 20 µg for nuclear fractions, were separated through electrophoresis on SDS–polyacrylamide gels and transferred to PVDF membranes using the Trans-Blot Turbo system (Bio-Rad, Madrid, Spain). Membranes were then blocked with 5% skim milk (*w*/*v*) in TBS 1× Tween (TBST 1×) and incubated overnight at 4 °C with primary antibodies (dilution 1:1000) against rabbit monoclonal antibody for LC3 (Cell Signaling, Amersham, Cytiva, Barcelona, Spain), mouse monoclonal antibody for Rev-erbα (Santa Cruz Biotechnology, Heidelberg, Germany), rabbit monoclonal antibody for Bmal1 (Cell Signaling, Amersham, Cytiva, Barcelona, Spain), rabbit monoclonal antibody for p62 (Cell Signaling, Amersham, Cytiva, Barcelona, Spain), rabbit monoclonal antibody for total AMPK (Cell Signaling, Amersham, Cytiva, Barcelona, Spain), rabbit monoclonal antibody for total pAMPK (Cell Signaling, Amersham, Cytiva, Barcelona, Spain), mouse monoclonal antibody for HO-1 (Santa Cruz Biotechnology, Heidelberg, Germany), rabbit monoclonal antibody for pNRF2 (#ab76026; Abcam, Cambridge, UK), mouse monoclonal antibody for NRF2 (Santa Cruz Biotechnology, Heidelberg, Germany), mouse monoclonal antibody for β-actin (Thermo Fisher Scientific, Madrid, Spain) and mouse monoclonal antibody for Histone 3 (H3) (Santa Cruz Biotechnology, Heidelberg, Germany). After washing, the membranes were incubated with HRP-conjugated secondary antibodies (dilution: 1:2000). Immunoreactive proteins were visualized using the ECL SelectTM Western Blotting Detection Reagent (Amersham ECL Select, Cytiva, Barcelona, Spain). Digital images were obtained with a G:BOX Chemi XL1.4 (Syngene, Cambridge, UK), and densitometric analyses were evaluated with Image Lab Software version 6.1 (Bio-Rad Laboratories, Hercules, CA, USA). Finally, the β-actin densitometric signal was used for normalization of total protein lysates, whereas H3 was used to normalize nuclear targets.

### 2.5. Gene Expression Analysis

A 20–30 mg fragment of liver tissue was mixed with TRIzol^®^ reagent (Thermo Fisher, Madrid, Spain) and homogenated with a Tissue Lyser LT (Qiagen, Madrid, Spain). After centrifugation, the supernatant was collected, and total RNA was extracted, including phase separation with chloroform and precipitation with isopropanol. The RNA pellet was resuspended with 60 µL of nuclease-free water (Thermo Fisher, Madrid, Spain). RNA concentration (ng/µL) and purity were determined using a NanoDrop ND-1000 spectrophotometer (Thermo Fisher, Madrid, Spain).

To study the gene expression of the liver samples, complementary DNA was produced by reverse transcription reaction of the extracted RNA with a High-Capacity cDNA Reverse Transcription kit (Thermo Fisher, Madrid, Spain). Quantitative polymerase chain reactions (qPCRs) of the obtained cDNAs were carried out in 384-well plates in a QuantStudio 5 Real-Time PCR System (Thermo Fischer, Madrid, Spain) using iTaq™ Universal SYBR^®^ Green Supermix (Bio-Rad, Barcelona, Spain). The normalization of the studied liver genes was done with the housekeeping gene peptidylprolyl isomerase A (*Ppia*). The primers used for each gene were purchased from Biomers.net (Ulm, Germany), specified in Table 1. Finally, the relative expression of each gene was determined using the 2^−ΔΔCt^ method, as reported by Schmittgen and Livak [27].

### 2.6. Metabolomic Analysis

Metabolomic analysis of the 48 rat liver samples was performed at the Centre for Omic Sciences (COS, Tarragona, Spain) using gas chromatography coupled with quadrupole time-of-flight mass spectrometry (GC-qTOF model 7200; Agilent, Santa Clara, CA, USA), as previously described [28,29]. Briefly, liver metabolomic analysis was performed using GC-qTOF following extraction with methanol:water (8:2) containing internal standards. Samples were homogenized and centrifuged, and supernatants were dried prior to derivatization (methoximation and silylation). Derivatized compounds were analyzed using the Fiehn method, with chromatographic separation on an HP5-MS column (Agilent Technologies; Santa Clara, CA, USA) and helium as the carrier gas. Detection was carried out by electron impact ionization (70 eV) in full-scan mode. Metabolites were identified by matching mass spectra and retention times with commercial standards and the Fiehn library (>1400 metabolites). Finally, metabolites were semi-quantified relative to internal standards.

### 2.7. Liver Lipid Profiling

Hepatic lipids were extracted using the Bligh–Dyer method [27]. Total cholesterol, triacylglycerol (TAG), and total lipid content in the liver were quantified with commercial colorimetric assay kits (QCA, Barcelona, Spain).

### 2.8. Statistical Analysis

Statistical analyses were performed using GraphPad Prism v. 8.0.1 software (GraphPad Software, San Diego, CA, USA), considering a *p*-value < 0.05 as statistically significant. Principal component analyses (PCAs) were performed using MetaboAnalyst 6.0 (https://www.metaboanalyst.ca/ accessed on 15 March 2026). Additionally, circadian rhythms in gene and protein expression profiles were assessed using the Cosinor-based rhythmometry method, which models oscillations and estimates rhythmic parameters. This statistical method models oscillatory patterns and provides rhythmic parameters that describe their characteristics. These include the MESOR (Midline Estimating Statistic of Rhythm), which represents the rhythm-adjusted mean; the amplitude, defined as half the difference between the peak and trough values; and the acrophase, corresponding to the time point at which the peak of the oscillation occurs. Analyses were performed with a Python version 3.7.4 script (v.2018.2.4; JetBrains s.r.o., Prague, Czech Republic) using the CosinorPy package (v.1.1) (Ljubljana, Slovenia) [30]. A circadian rhythm was assumed when the expression model fit a curve with a *p*-value < 0.05.

## 3. Results

### 3.1. CAF Diet Effects on Hepatic Circadian Clock

**Figure 1:**
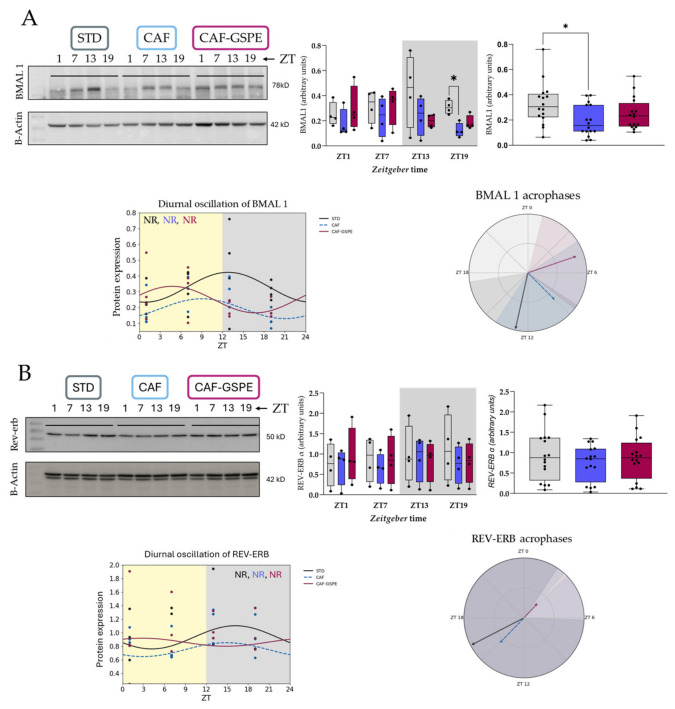
Effect of CAF diet and GSPE supplementation on BMAL1 and REV-ERBα proteins at different time points. Brain and muscle Arnt-like protein-1 (BMAL1) (**A**) and REV-ERBα (**B**) were analyzed in 48 male Fischer 344 rats fed an STD diet or CAF diet with daily supplementation of either vehicle or GSPE, administered at the onset of the dark phase (ZT12). Rats were sacrificed at four different ZTs: 9 a.m. (ZT1), 3 p.m. (ZT7), 9 p.m. (ZT13), and 3 a.m. (ZT19). Top left panels show representative immunoblots for BMAL1 and REV-ERBα (*n* = 4). Bottom left panels display diurnal protein expression profiles for BMAL1 and REV-ERBα (*n* = 4). Top right panels present Western blot quantification statistics expressed as minimum to maximum values, medians, and interquartile ranges (*n* = 4) at each time point and (*n* = 16) for each diet/treatment condition. Bottom left panels illustrate acrophases and amplitudes of BMAL1 and REV-ERBα expression for STD-VH, CAF-VH, and CAF-GSPE groups, with rhythm parameters determined using CircAnalyst v1.0. Statistical significance in immunoblots is indicated as follows: * *p* < 0.05 significant differences due to CAF diet.

To assess whether the CAF diet alters hepatic circadian regulation and whether GSPE supplementation can modulate these effects, the protein expression of the core clock components BMAL1 and REV-ERBα was analyzed across four Zeitgeber times (ZTs). Regarding absolute protein abundance, BMAL1 expression was significantly reduced depending on the intervention, with the CAF-VH group showing lower levels compared to STD-VH rats, specifically at ZT19 (Mann–Whitney test, *p* = 0.0286; Figure 1). GSPE supplementation did not significantly reverse this CAF-induced reduction, although a trend toward partial recovery was observed. In contrast, REV-ERBα protein levels were similar across all diet-treatment groups, showing no statistically significant differences at any ZT (Figure 1).

Beyond absolute protein levels, circadian rhythm analysis confirmed that the CAF diet severely disrupted the temporal organization of these core clock components. While BMAL1 protein lost its rhythmic expression pattern under obesogenic conditions (Table S3), GSPE supplementation failed to fully restore its global oscillatory parameters, showing only a significant amplitude and a shift in acrophase without reaching overall rhythmicity (Supplementary Tables S2 and S3). Similarly, REV-ERBα lacked robust circadian rhythmicity across all experimental groups, and its temporal profile remained largely unaffected by either diet or treatment. The detailed statistical parameters for these rhythmic oscillations are provided in Supplementary Note S1.

In summary, these findings indicate that obesogenic feeding is associated with a marked alteration of the central hepatic clock, reflected by reduced BMAL1 protein levels and a disruption of its temporal organization. GSPE supplementation did not fully restore these alterations, suggesting a limited impact on core clock components. This observation provides a framework for interpreting its effects at other regulatory levels, supporting the possibility that GSPE may act through downstream pathways to modulate metabolic and redox responses under obesogenic conditions.

### 3.2. Effect of the CAF Diet and GSPE Supplementation on the Circadian Regulation of Hepatic Autophagy

**Figure 2:**
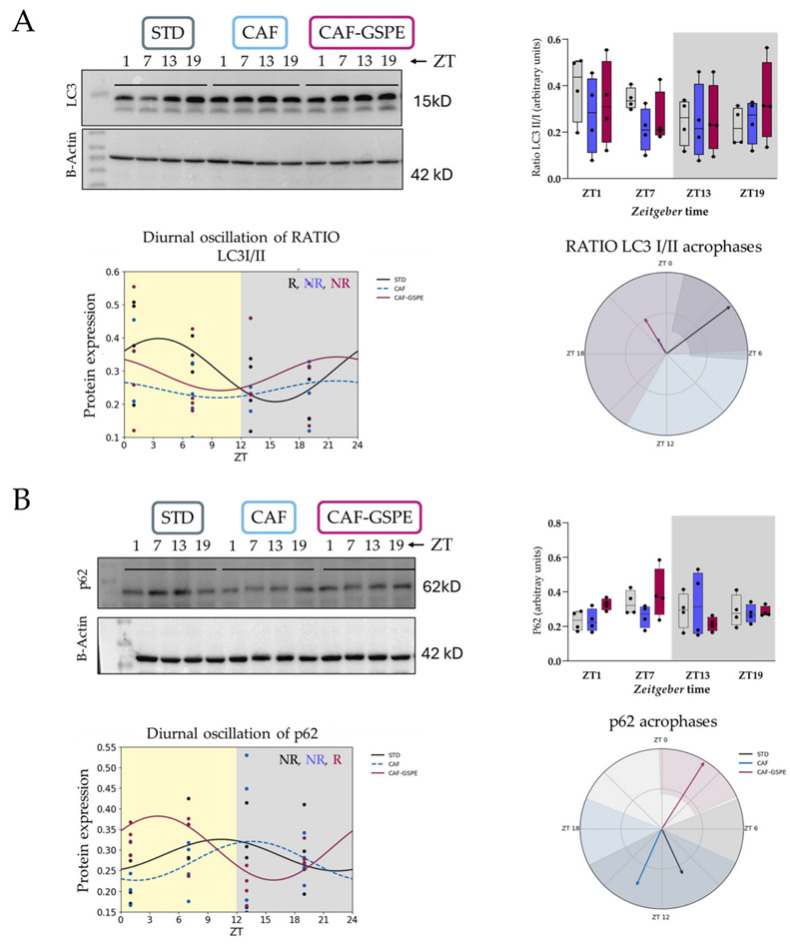
Effect of CAF diet and GSPE supplementation on LC3 and p62 proteins at different time points. Microtubule-associated protein 1A/1B-light chain 3 (LC3) (**A**) and Sequestosome 1 (SQSTM1 or p62) (**B**) were analyzed in 48 male Fischer 344 rats fed an STD diet or CAF diet with daily supplementation of either vehicle or GSPE, administered at the onset of the dark phase (ZT12). Rats were sacrificed at four different ZTs: 9 a.m. (ZT1), 3 p.m. (ZT7), 9 p.m. (ZT13), and 3 a.m. (ZT19). Top left panels show representative immunoblots for LC3 and p62 proteins (*n* = 4). Bottom left panels display diurnal protein expression profiles for LC3 and p62 proteins (*n* = 4). Top right panels present Western blot quantification statistics expressed as minimum to maximum values, medians, and interquartile ranges (*n* = 4). Bottom right panels illustrate acrophases and amplitudes of LC3 and p62 protein expression for STD-VH, CAF-VH, and CAF-GSPE groups, with rhythm parameters determined using CircAnalyst. Rhythmicity is expressed as “R” and no rhythmicity is expressed as “NR”.

To investigate the impact of the CAF diet on hepatic autophagy and the potential modulatory effects of GSPE supplementation, the expression of key autophagic markers, LC3 and p62, at both protein and transcript levels was analyzed. Regarding absolute protein content, no statistically significant differences were observed in the LC3-II/I ratio or p62 protein levels across the diet-treatment groups at any specific ZT according to the Mann–Whitney test (Figure 2).

Despite the lack of significant changes in absolute total protein levels, circadian rhythm analysis revealed that CAF feeding disrupted the temporal rhythmic organization of these key autophagic markers. The LC3-II/I ratio displayed a tendency toward circadian rhythmicity in STD-VH rats (*p* = 0.050), whereas both the CAF-VH and CAF-GSPE groups were completely arrhythmic (*p* = 0.881 and *p* = 0.650, respectively). In STD-VH rats, the ratio peaked at ZT4, while in both CAF-fed groups, the peak was shifted to ZT22. For p62, while the STD-VH and CAF-VH groups lacked statistically significant rhythmicity (*p* = 0.461 and *p* = 0.470, respectively), GSPE supplementation implemented a significant diurnal rhythm in CAF-GSPE livers (*p* = 0.0146). Peak p62 levels occurred at ZT10 in STD-VH, ZT14 in CAF-VH, and ZT2 in CAF-GSPE livers.

**Figure 3:**
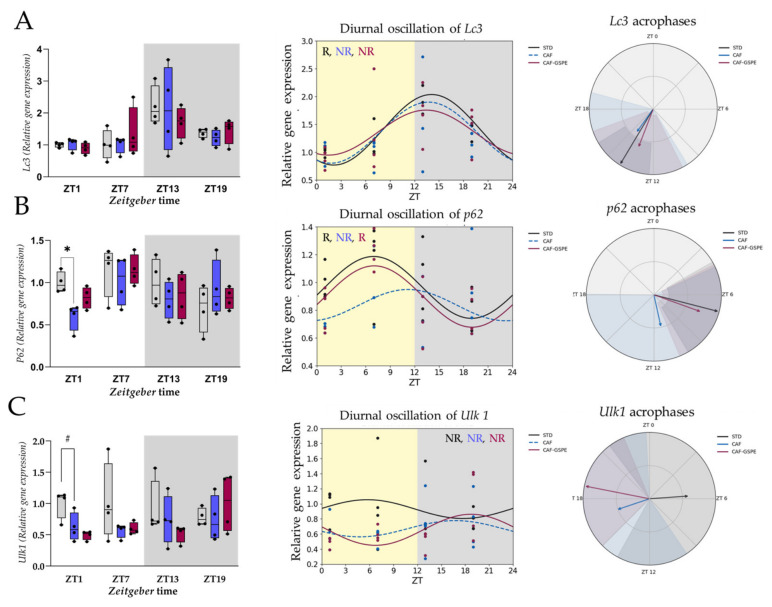
CAF and GSPE effects on LC3 (**A**), p62 (**B**), and ULK1 (**C**) gene expression at different time points. Microtubule-Associated Protein 1 Light Chain 3 (LC3) (**A**) and Sequestosome 1 (SQSTM1 or p62) (**B**) were analyzed in 48 male Fischer 344 rats that were fed with an STD or CAF diet and received a daily supplementation of vehicle or GSPE at the beginning of the activity/dark phase (ZT12) and were sacrificed at different time points: 9 a.m. (ZT1), 3 p.m. (ZT7), 9 p.m. (ZT13) and 3 a.m. (ZT19). (**left**) RT-qPCR statistics for LC3, p62 and ULK1 at different time points (*n* = 4). (**middle**) Diurnal gene expression profile for LC3 gene (*n* = 4). (**right**) Acrophase and amplitude represented for LC3, p62 and ULK1 expression for the STD-VH, CAF-VH and CAF-GSPE groups. For “(**left**)”, data are expressed as minimum to maximum values, medians and interquartile ranges. For “(**middle**)”, data are expressed as means ± SEMs, and the determination of rhythm parameters was done with CircAnalyst. “#” *p* > 0.05 indicates tendency as determined by Mann–Whitney test. “*” *p* < 0.05 indicates significant differences as determined by Mann–Whitney test.

Similarly, at the transcriptional level, *Lc3* expression followed a circadian rhythm in STD-VH rats (*p* = 0.005) but was completely arrhythmic in CAF-VH and CAF-GSPE rats (*p* = 0.333 and *p* = 0.110, respectively). Regarding *p62*, gene expression was significantly downregulated in CAF-VH livers compared to STD-VH, specifically at ZT1 (*p* = 0.0286; Figure 3). Across other time points, *p62* transcript levels remained largely unchanged, and GSPE treatment did not induce significant absolute effects. However, circadian analysis of *p62* suggested a tendency toward rhythmicity in STD-VH and CAF-GSPE livers (*p* = 0.077 and *p* = 0.098, respectively), whereas CAF-VH livers were entirely arrhythmic (*p* = 0.566). Although GSPE supplementation partially restored specific oscillatory features, recovering significant amplitude and acrophase for *p62* and *Lc3*, several rhythmic parameters remained decoupled from the standard physiological state. Furthermore, Ulk1 gene expression tended to be lower in CAF-VH livers at ZT1 compared to STD-VH (*p* = 0.0571, Mann–Whitney test), while GSPE supplementation did not significantly alter Ulk1 transcript levels relative to CAF-VH (Figure 3). The detailed statistical parameters for these rhythmic oscillations are provided in Supplementary Note S3.

Overall, the CAF diet was associated with a disruption of the temporal regulation of key autophagy-related markers at both protein and transcript levels. GSPE supplementation partially restored selected oscillatory features, particularly for p62 and LC3, although several rhythmic parameters remained altered compared to STD-VH animals. These observations suggest that obesogenic feeding impairs the temporal coordination of hepatic autophagy, while GSPE may contribute to a partial reorganization of these dynamics. Rather than fully re-establishing physiological rhythmicity, GSPE appears to modulate specific aspects of autophagic regulation, potentially improving the alignment between cellular quality-control processes and metabolic demands under obesogenic conditions.

### 3.3. CAF Diet Effects on Hepatic Antioxidant Responses (NRF2/HO-1 Axis)

**Figure 4:**
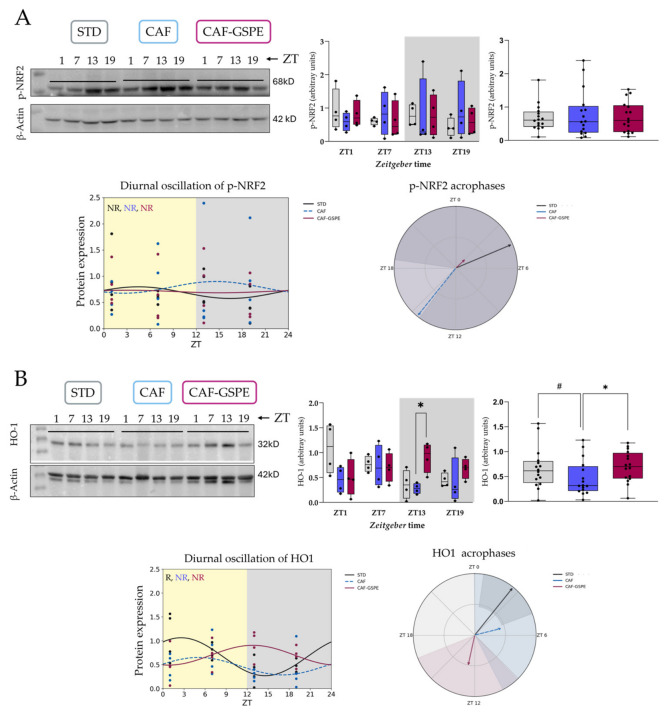
Effect of CAF diet and GSPE supplementation on NRF2/HO-1 axis at different time points. Phosphorylated Nuclear factor erythroid 2-related factor 2 (p-NRF2) (**A**), Heme oxygenase 1 (HO-1) (**B**), and Phosphorylated Nuclear factor erythroid 2–related factor 2 (p-NRF2) were analyzed in 48 male Fischer 344 rats fed an STD diet or CAF diet with daily supplementation of either vehicle or GSPE, administered at the onset of the dark phase (ZT12). Rats were sacrificed at four different ZTs: 9 a.m. (ZT1), 3 p.m. (ZT7), 9 p.m. (ZT13), and 3 a.m. (ZT19). Top left panels show representative immunoblots for p-NRF2 and HO-1 proteins (*n* = 4). Bottom left panels display diurnal protein expression profiles for p-NRF2 and HO-1 proteins (*n* = 4). Top right panels present Western blot quantification statistics expressed as minimum to maximum values, medians, and interquartile ranges (*n* = 4) at each time point and (*n* = 16) for each diet/treatment condition. Bottom right panels illustrate acrophases and amplitudes of p-NRF2 and HO-1 protein expression for STD-VH, CAF-VH, and CAF-GSPE groups, with rhythm parameters determined using CircAnalyst. Rhythmicity is expressed as “R” and no rhythmicity is expressed as “NR”. * *p* < 0.05 for signicance differences; # *p* > 0.05 for statistical tendency (Mann–Whitney test).

To evaluate whether CAF feeding disrupts hepatic redox regulation and whether GSPE supplementation can modulate these effects, the protein expression of phosphorylated NRF2 (pNRF2) and its downstream target, heme oxygenase-1 (HO-1), was analyzed across four Zeitgeber times (ZTs). Consequently, regarding absolute protein levels, pNRF2 showed a general qualitative increase at ZT13 across all experimental groups; however, no statistically significant differences were observed between diet or treatment conditions (Figure 4A). In contrast, HO-1 protein expression displayed a strong differential response depending on the intervention. Obesogenic feeding in CAF-VH rats altered HO-1 levels compared to STD-VH animals, indicating an impaired antioxidant capacity under metabolic stress. Notably, GSPE supplementation effectively counteracted this impairment by enhancing HO-1 protein expression. This potentiation of antioxidant defenses was particularly pronounced and targeted at ZT13, where HO-1 levels were significantly higher than those observed in untreated CAF-VH rats (Mann–Whitney test, *p* = 0.0288; Figure 4B).

To further characterize this time-dependent response, a comprehensive circadian rhythm analysis was performed (Supplementary Note S4 and Tables S8 and S9). While pNRF2 lacked statistical 24 h rhythmicity across all experimental groups, HO-1 exhibited a significant physiological circadian rhythm in healthy rats (*p* = 0.010) that was completely abolished by the CAF diet. Importantly, rather than fully restoring the baseline 24 h oscillation, GSPE supplementation induced a highly targeted temporal pattern. It drove a significant amplitude and a distinct acrophase peaking closely to ZT13 (*p* < 0.001), confirming that the extract exerts a time-specific, chrono-pharmacological enhancement of antioxidant defenses exactly at the onset of the active phase.

**Figure 5:**
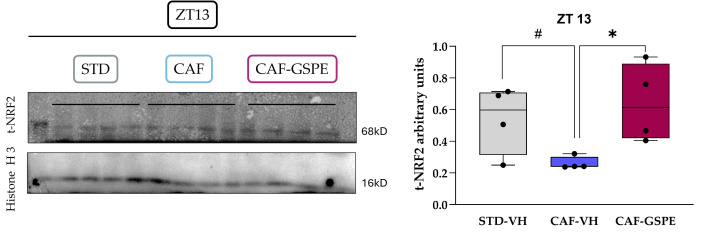
Effect of CAF diet and GSPE supplementation on nuclear NRF2 accumulation at ZT13. Total nuclear NRF2 (t-NRF2) protein levels were determined in isolated hepatic nuclear fractions from male Fischer 344 rats fed a standard diet (STD-VH) or cafeteria diet (CAF-VH), with or without GSPE supplementation (CAF-GSPE). ZT13 was selected because it corresponded to the time point at which GSPE induced the maximal HO-1 response. Representative immunoblots and densitometric quantification normalized to Histone H3 are shown. Data are expressed as minimum to maximum values, medians, and interquartile ranges (*n* = 4 per group). * *p* < 0.05 vs. CAF-VH; # *p* > 0.05 vs. STD-VH (Mann–Whitney test).

To specifically explore whether this time-specific HO-1 induction could be associated with canonical NRF2 activation, we evaluated total NRF2 (t-NRF2) protein levels in isolated hepatic nuclear fractions at this critical temporal window (ZT13) (Figure 5). In obesogenic conditions, nuclear t-NRF2 accumulation tended to be lower in the CAF-VH group compared with healthy controls (STD-VH, *p* = 0.0571), suggesting a blunted NRF2 nuclear response at the onset of the active phase. GSPE supplementation significantly increased nuclear t-NRF2 levels compared with CAF-VH rats (*p* = 0.0286), restoring values close to those of STD-VH animals. Taken together, these results support the notion that the chrono-selective induction of HO-1 at ZT13 by GSPE is accompanied by a time-specific recovery of NRF2 nuclear accumulation, consistent with, but not definitively proving, canonical NRF2 activation.

### 3.4. CAF Diet and GSPE Effects on AMPK Activation

**Figure 6:**
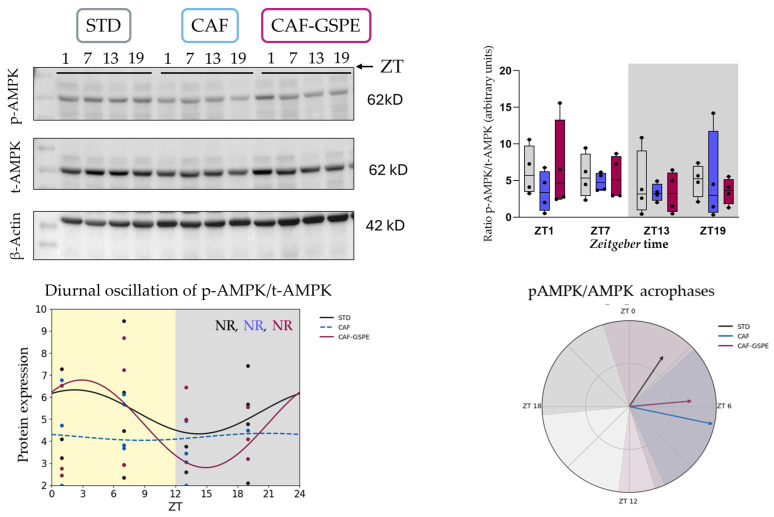
Effect of CAF and GSPE supplementation on AMPK activity at different time points. Forty-eight male Fischer 344 rats were fed with an STD or CAF diet and received a daily supplementation of vehicle or GSPE at the beginning of the activity/dark phase (ZT12) and were sacrificed at different time points: 9 a.m. (ZT1), 3 p.m. (ZT7), 9 p.m. (ZT13) and 3 a.m. (ZT19). Top left panels show representative immunoblots for AMPK (*n* = 4). Top right panels display diurnal protein expression profiles. Top right panels present Western blot quantification statistics expressed as minimum to maximum values, medians, and interquartile ranges (*n* = 4). Bottom right panels illustrate acrophases and amplitudes, with rhythm parameters determined using CircAnalyst. Rhythmicity is expressed as “R” and no rhythmicity is expressed as “NR”.

Given the central role of AMPK as a master regulator of cellular energy homeostasis and an upstream initiator of autophagy, we evaluated its activation status by measuring the phosphorylated-to-total AMPK (pAMPK/AMPK) ratio across the 24 h cycle (Figure 6). Regarding absolute activation levels, a qualitative dampening of AMPK phosphorylation was observed in the CAF-VH group relative to both the healthy controls (STD-VH) and the GSPE-treated animals. However, these variations remained modest and did not reach statistical significance at any specific Zeitgeber time (ZT). Furthermore, circadian rhythm analysis revealed that AMPK activation did not exhibit a statistically robust 24 h oscillatory pattern in any of the experimental conditions (*p* = 0.692 for STD-VH, *p* = 0.193 for CAF-VH, and *p* = 0.529 for CAF-GSPE). Although peak activation ratios were observed at ZT2 in STD-VH, ZT7 in CAF-VH, and ZT6 in CAF-GSPE rats, the high inter-individual variability prevented the detection of a statistically significant rhythmic pattern.

### 3.5. CAF Diet and GSPE Effects on ER Stress-Related Genes

**Figure 7:**
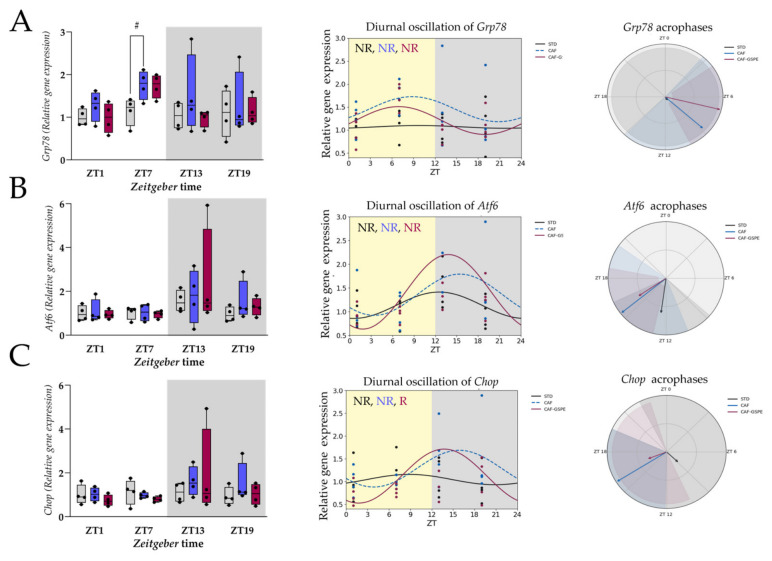
CAF and GSPE effects on *Grp78* (**A**), *Atf6* (**B**), and *Chop* (**C**) gene expression at different time points. Glucose-Regulated Protein 78 (*Grp78*), Activating Transcription Factor 6 (*Atf6*), and DNA damage-inducible transcript 3 (*Chop*) were analyzed in 48 male Fischer 344 rats that were fed with an STD or CAF diet and received a daily supplementation of vehicle or GSPE at the beginning of the activity/dark phase (ZT12) and were sacrificed at different time points: 9 a.m. (ZT1), 3 p.m. (ZT7), 9 p.m. (ZT13) and 3 a.m. (ZT19). From left to right: RT-qPCR analysis at different time points (*n* = 4), diurnal gene expression profiles (*n* = 4), and acrophase and amplitude of expression in the STD-VH, CAF-VH, and CAF-GSPE groups. In the first panel, data are presented as minimum to maximum values, medians, and interquartile ranges. In the second panel, data are expressed as means ± SEMs. Rhythm parameters were determined using CircAnalyst. “#” indicates *p* > 0.05, showing a statistical trend for the effect of STD diet and CAF diet according to the Mann–Whitney test. Rhythmicity is expressed as “R” and no rhythmicity is expressed as “NR”.

To evaluate whether the observed metabolic and autophagic disruptions were associated with a global induction of endoplasmic reticulum (ER) stress, the transcript levels of key unfolded protein response (UPR) mediators, *Chop* and *Atf6*, were analyzed. Interestingly, the absolute expression levels of both markers remained largely stable across the 24 h cycle in all experimental conditions. No statistically significant differences in *Chop* or *Atf6* expression were observed between healthy controls (STD-VH) and CAF-fed animals, nor between the untreated and GSPE-supplemented CAF groups at any specific Zeitgeber time (Figure 7).

In this regard, the absence of a pronounced diet-induced upregulation in these classical ER stress genes could be considered as a highly informative finding because it could suggest that the circadian dysregulation and autophagic impairment driven by the obesogenic diet in this model are not the secondary consequence of a massive, generalized UPR overload. Furthermore, it likely demonstrates that the chrono-pharmacological rescue exerted by GSPE, particularly the targeted enhancement of the HO-1 antioxidant defense, operates through highly specific redox and metabolic pathways, rather than through a broad modulation of the ER stress response.

To further explore potential temporal modulations within the UPR pathway, a detailed circadian rhythm analysis was conducted (Supplementary Note S5 and Tables S10 and S11). Consistent with the stable absolute expression levels, robust physiological 24 h rhythmicity for these ER stress markers was largely absent in both the control and CAF-fed animals. While GSPE supplementation induced minor temporal realignments—such as establishing a significant diurnal rhythm for *Atf6* (*p* = 0.049)—neither the obesogenic diet nor the extract provoked major disruptions or global restorations in the rhythmic parameters of this pathway. These chronobiological data reinforce the concept that the metabolic benefits of GSPE are highly targeted. By effectively bypassing the generalized ER stress machinery, GSPE acts directly on specific downstream antioxidant and autophagic networks to counteract obesogenic damage.

### 3.6. Lipid Liver Profile

A clear CAF effect was observed in the hepatic triglyceride, cholesterol and total lipid levels, which were significantly higher in CAF-fed rats compared with those on the standard diet (*p* = 0.0286). In addition, GSPE supplementation reduced the triglyceride (TAG) content in comparison with the CAF group (*p* = 0.0286) (Table 2). Total cholesterol was also significantly increased in CAF-fed animals compared with STD-fed rats. In contrast, total liver lipid contents showed no significant differences among groups.

### 3.7. Liver Metabolomics Profiles

In order to investigate the impact of dietary challenge and GSPE supplementation on hepatic metabolism, we conducted a comprehensive metabolomic analysis and multivariate statistical analysis. This analysis identified and quantified 66 metabolites across all experimental groups.

#### 3.7.1. Global Metabolic Structure and Circadian Organization

Principal component analysis (PCA) revealed differences in liver metabolite profiles between ZTs in all our diet/treatment groups. Permutational multivariate analysis of variance (PERMANOVA) confirmed significant temporal separation in the STD-VH (*p* = 0.003) and CAF-GSPE (*p* = 0.021) groups, whereas CAF-fed animals showed a non-significant trend toward clustering (*p* = 0.066).

**Figure 8:**
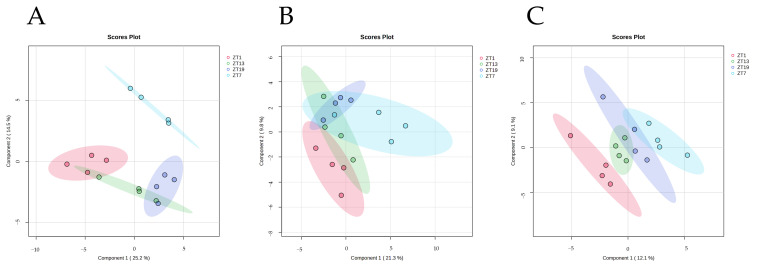
Dietary impact on the temporal organization of the hepatic metabolome. Sparse Partial Least Squares Discriminant Analysis (sPLS-DA) score plots showing hepatic metabolic clustering for: (**A**) STD-VH, (**B**) CAF-VH and (**C**) CAF-GSPE. Each panel represents the distribution of hepatic profiles across four ZTs: ZT1 (red), ZT7 (green), ZT13 (blue), and ZT19 (purple). The separation along the first two components (Comp 1 and Comp 2) reflects the daily rhythmic oscillations of the liver metabolome. Shaded areas denote 95% confidence ellipses. Temporal clustering was assessed by PERMANOVA (*p* = 0.003, *p* = 0.066, and *p* = 0.021 for (**A**), (**B**), and (**C**), respectively). Note the loss of distinct temporal separation in the CAF-VH group and its partial recovery with GSPE supplementation.

Partial Least Squares Discriminant Analysis (PLS-DA) further illustrated these differences (Figure 8). In STD-fed animals, samples clustered clearly according to circadian time (ZT1, ZT7, ZT13, and ZT19), reflecting a preserved daily oscillatory organization (Figure 8A). In contrast, CAF feeding resulted in a marked overlap between time points and a loss of distinct temporal clustering (Figure 8B). Notably, GSPE supplementation improved the separation of metabolic profiles by ZT in CAF-fed rats, indicating a partial restoration of the temporal metabolomic structure (Figure 8C).

#### 3.7.2. Identification of Discriminant Metabolites

PLS-DA identified 18 metabolites with variable importance in projection (VIP) scores ≥ 1.0, indicating major contributions to group separation (Table 3). These metabolites were mainly related to amino acid, tricarboxylic acid (TCA) cycle, glucose, and lipid metabolism. Among them, glutamine, pyruvic acid and cholesterol showed the highest VIP values in the STD-VH, CAF-VH and CAF-GSPE groups, respectively, suggesting strong discriminant power between groups. Table 3 highlights the ranking of metabolites according to VIP scores.

#### 3.7.3. Metabolic Pathway Representation

**Figure 9:**
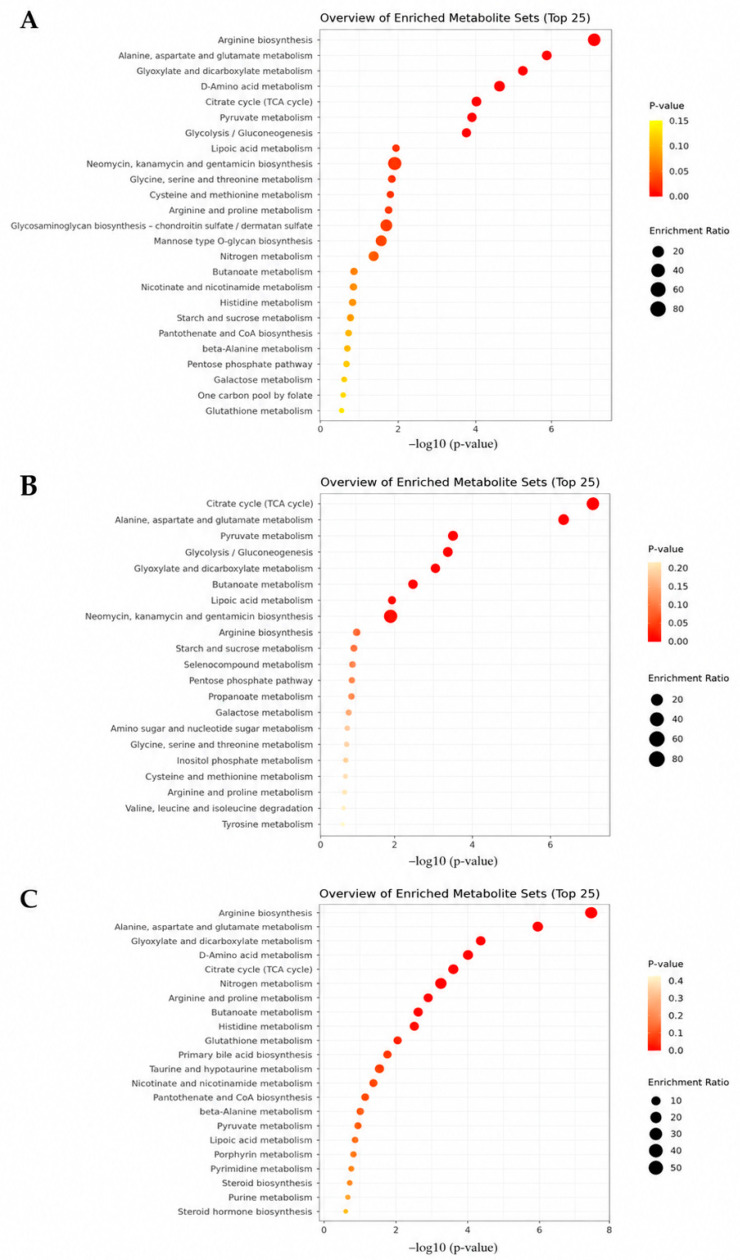
Metabolite Set Enrichment Analysis (MSEA) of differentially regulated metabolites. Bubble plots representing the top 25 enriched metabolic pathways based on the hepatic metabolic profiles of rats fed with: (**A**) standard diet (STD-VH), (**B**) cafeteria diet (CAF-VH), and (**C**) cafeteria diet supplemented with grape seed proanthocyanidin extract (CAF+GSPE).

Discriminant metabolites belonged predominantly to amino acid metabolism (glutamine, serine, ornithine, aspartate, alanine, glutamate, and taurine), the TCA cycle (α-ketoglutarate, malate, citrate, and succinate), glucose metabolism (glucose-6-phosphate, pyruvate, and lactate), and lipid metabolism (glycerol-1-phosphate, 3-hydroxybutyric acid, and cholesterol), together with xylonic acid (Table 3). Finally, KEGG pathway enrichment analysis (Figure 9 and Table S12) was performed to identify metabolic pathways significantly represented within the metabolite sets associated with each experimental condition. Pathways with a false discovery rate (FDR) < 0.05 were considered statistically significant. In animals fed a standard diet (STD), enrichment analysis revealed a metabolic profile characterized by coordinated activity of amino acid metabolism and central carbon metabolism pathways. The most significantly enriched pathway was arginine biosynthesis (four hits; FDR = 4.5 × 10^−5^), followed by alanine, aspartate and glutamate metabolism (four hits; FDR = 4.4 × 10^−4^) and glyoxylate and dicarboxylate metabolism (four hits; FDR = 5.1 × 10^−4^). Additional significantly enriched pathways included D-amino acid metabolism (three hits; FDR = 1.0 × 10^−3^), the citrate cycle (TCA cycle) (three hits; FDR = 2.5 × 10^−3^), pyruvate metabolism (three hits; FDR = 2.8 × 10^−3^), and glycolysis/gluconeogenesis (three hits; FDR = 3.2 × 10^−3^). Together, these pathways indicate that under physiological conditions the hepatic metabolite profile reflects a balanced integration of amino acid metabolism and mitochondrial energy pathways.

In contrast, CAF-fed animals displayed a markedly different enrichment pattern dominated by pathways related to central carbon metabolism. The most significantly enriched pathway was the citrate cycle (TCA cycle) (five hits; FDR = 2.29 × 10^−6^), followed by alanine, aspartate and glutamate metabolism (five hits; FDR = 7.12 × 10^−6^). Additional enriched pathways included pyruvate metabolism (three hits; FDR = 5.55 × 10^−3^), glycolysis/gluconeogenesis (three hits; FDR = 5.55 × 10^−3^), and glyoxylate and dicarboxylate metabolism (three hits; FDR = 1.06 × 10^−2^). Butanoate metabolism was also significantly enriched (two hits; FDR = 4.25 × 10^−2^). Overall, CAF feeding was therefore associated with a metabolomic profile characterized by strong enrichment of glycolysis, pyruvate metabolism and mitochondrial TCA cycle pathways, suggesting increased metabolic flux through central carbon metabolism.

In animals receiving GSPE supplementation, enrichment analysis revealed a metabolic profile distinct from that observed in CAF-fed animals and dominated primarily by pathways related to amino acid metabolism and nitrogen handling. The most significantly enriched pathway was arginine biosynthesis (five hits; FDR = 5.95 × 10^−7^), followed by alanine, aspartate and glutamate metabolism (five hits; FDR = 1.41 × 10^−5^). Other significantly enriched pathways included glyoxylate and dicarboxylate metabolism (four hits; FDR = 8.44 × 10^−4^), D-amino acid metabolism (three hits; FDR = 1.46 × 10^−3^), the citrate cycle (TCA cycle) (three hits; FDR = 3.58 × 10^−3^), and nitrogen metabolism (two hits; FDR = 7.79 × 10^−3^). Additional enriched pathways comprised arginine and proline metabolism, butanoate metabolism, and histidine metabolism (FDR < 0.05).

Comparison of enrichment profiles across experimental groups revealed clear metabolic differences between dietary conditions. While STD animals displayed enrichment of both amino acid metabolism and central carbon pathways, CAF feeding resulted in a pronounced enrichment of glycolysis, pyruvate metabolism and TCA cycle pathways, indicating a shift toward increased substrate flux through central energy metabolism. In contrast, GSPE supplementation shifted the enrichment landscape toward pathways associated with amino acid metabolism and nitrogen handling, including arginine biosynthesis and alanine–aspartate–glutamate metabolism. Notably, several pathways linked to cellular redox and nitrogen metabolism were preferentially represented in GSPE-treated animals. In total, seven pathways were significantly enriched in STD animals, six in CAF animals, and nine in GSPE-treated animals (FDR < 0.05).

Taken together, these findings indicate that CAF feeding promotes a metabolic profile dominated by central carbon metabolism and mitochondrial energy pathways, whereas GSPE supplementation is associated with enrichment of pathways related to amino acid metabolism, nitrogen handling and cellular stress adaptation. These metabolomic signatures provide a metabolic framework consistent with the biochemical and molecular alterations observed in the liver, including changes in antioxidant defenses and stress-response pathways. In particular, the enrichment of amino acid and redox-related metabolic pathways in GSPE-treated animals may contribute to the restoration of hepatic redox homeostasis observed in parallel analyses of antioxidant signaling.

## 4. Discussion

Metabolic dysfunction-associated steatotic liver disease (MASLD) is increasingly recognized not merely as a disorder of lipid accumulation, but as a systemic disruption of hepatic metabolic homeostasis involving redox imbalance, impaired autophagy, and altered temporal coordination of metabolic processes [1,2]. Recent evidence further supports the notion that obesogenic environments destabilize peripheral circadian oscillators and uncouple metabolic fluxes from physiological daily rhythms, thereby promoting metabolic inflexibility and disease progression [2,3,31]. In line with this, our data indicate that cafeteria (CAF) feeding is associated with a marked disruption of hepatic temporal organization, supporting the concept of chrono-metabolic misalignment under chronic nutrient overload.

At the mechanistic level, this disruption appears to involve alterations in the core molecular clock. BMAL1 plays a central role in coordinating hepatic nutrient utilization with feeding–fasting cycles, mitochondrial function, and redox homeostasis [4,32]. Reduced BMAL1 expression, as observed in VH-CAF-fed rats, has been associated with mitochondrial dysfunction, altered lipid handling, and increased susceptibility to steatosis [32]. This alteration may contribute to downstream disturbances in cellular quality-control systems, particularly autophagy, which is tightly regulated in a time-dependent manner to maintain metabolic balance [33]. Consistent with this, the loss of rhythmicity in LC3 and p62 observed here aligns with previous reports linking obesogenic diets to impaired lipophagy and defective lipid turnover [7,34].

Functionally, these alterations are associated with a metabolic phenotype characterized by accumulation of glycolytic and tricarboxylic acid (TCA) cycle intermediates, suggestive of mitochondrial overload and reduced oxidative capacity [35]. Under these conditions, p62 accumulation would be expected to activate NRF2 through KEAP1 sequestration [12,36], thereby promoting an adaptive antioxidant response. However, the reduced HO-1 expression observed in CAF-fed animals suggests that this compensatory mechanism is impaired, consistent with reports describing NRF2 dysfunction and redox imbalance in chronic metabolic stress [37]. This hypothesis is strongly supported by our target analysis of the hepatic nuclear fraction at ZT13, which revealed a clear trend toward blunted NRF2 nuclear accumulation under obesogenic conditions. Crucially, GSPE intervention significantly reversed this impairment, stimulating NRF2 nuclear localization at the exact temporal window where HO-1 expression peaks. By driving this time-specific rescue of the NRF2/HO-1 axis, GSPE contributes to a partial reorganization of these cellular dynamics.

Although HO-1 is classically considered an NRF2 target gene, its regulation is highly multifactorial and involves additional transcriptional regulators, including AP-1, ATF4, HIF-1α, NF-κB, and the transcriptional repressor Bach1. Moreover, increasing evidence supports extensive crosstalk between antioxidant defense pathways and circadian regulators, suggesting that HO-1 expression may also be subjected to temporal control. Therefore, the marked increase in HO-1 observed after GSPE administration, particularly at ZT13, may reflect a broader chrono-adaptive cytoprotective response rather than an exclusive NRF2-driven mechanism. Notably, the selective induction of HO-1 at ZT13 despite modest changes in pNRF2 further supports the possibility that GSPE may engage alternative regulatory pathways involved in HO-1 expression and circadian stress adaptation [38,39,40,41].

Interestingly, the absence of robust diet- or treatment-induced changes in global AMPK activation suggests that the temporal alterations observed in autophagic markers, as well as the effects associated with GSPE, may occur independently of major shifts in this energy sensor. Therefore, AMPK-related observations in the liver should be interpreted cautiously as exploratory and contextual rather than as evidence of robust pathway activation. Consequently, GSPE appears to exert its biological responses through mechanisms that are not primarily driven by AMPK signaling but are instead more closely associated with downstream modulations of autophagic pathways and the restoration of the NRF2/HO-1 redox axis under obesogenic conditions.

This mechanistic interpretation is further supported by the metabolomic profile observed in GSPE-treated animals. CAF feeding promotes a metabolic phenotype dominated by central carbon metabolism and mitochondrial energy pathways, whereas GSPE supplementation is associated with enrichment of pathways related to amino acid metabolism, nitrogen handling and cellular stress adaptation. This is supported by the observed shift toward amino acid-related metabolic pathways, including glutamine, taurine, and ornithine metabolism, which are known to support glutathione synthesis, mitochondrial function, and redox buffering capacity [21,34,42]. These metabolomic signatures provide a metabolic framework consistent with the biochemical and molecular alterations observed in the liver, including changes in antioxidant defenses and stress-response pathways. In particular, the enrichment of amino acid- and redox-related metabolic pathways in GSPE-treated animals may contribute to the restoration of hepatic redox homeostasis observed in parallel analyses of antioxidant signaling. The temporal specificity in GSPE-induced responses is consistent with the emerging concept of polyphenols as modulators of circadian and metabolic pathways, often referred to as “chrononutrients” [43,44].

Despite its findings, this study has limitations that should be acknowledged. First, the use of a single male Fischer 344 rat strain may limit the generalizability of our results, although it ensures consistency with our previous metabolic research. Second, while we have demonstrated a time-specific nuclear accumulation of t-NRF2 at ZT13, direct assessment of antioxidant response element (ARE) binding activity or downstream genetic validation would be required to definitively confirm canonical NRF2 transcriptional activation. Lastly, the inherently heterogeneous polyphenolic composition of GSPE introduces complexity when characterizing individual bioactive targets. To overcome these limitations, future research by our group or others should incorporate multi-strain and mixed-sex metabolic models as well as execute functional ARE-binding assays alongside broader antioxidant pathway analyses to fully map this exploratory signaling cascade.

From a translational perspective, these findings reinforce the clinical importance of considering circadian timing when designing nutritional interventions targeting oxidative stress and metabolic liver alterations. Specifically, the observed time-dependent effects of GSPE on hepatic redox homeostasis highlight the potential for developing innovative chrono-nutritional or chrono-pharmacological strategies based on natural bioactive compounds. Aligning the administration of such components with specific circadian phases of metabolic vulnerability could offer a novel, non-invasive therapeutic avenue for the management of obesity-associated metabolic dysfunction and related conditions like MASLD [43,44].

In conclusion, CAF feeding is associated with a disruption of hepatic chrono-metabolic organization, while GSPE supplementation appears to partially restore redox–metabolic coordination through time-dependent modulation of antioxidant defenses. Rather than acting as a global circadian synchronizer, GSPE may function as a chrono-selective modulator, enhancing specific antioxidant responses, particularly HO-1, in physiologically relevant time windows.

## Figures and Tables

**Table 1:** Nucleotide sequences of primers used for real-time quantitative PCR.

Gene	Accession Number (NCBI)	Forward Primer (5′ to 3′)	Reverse Primer (5′ to 3′)
*Grp78*	NM_013083	TCGACTTGGGGACCACCTAT	GCCCTGATCGTTGGCTATGA
*Atf6*	NM_001107196	GGACCAGGTGGTGTCAGAG	GACAGCTCTGCGCTTTGGG
*Chop*	NM_001109986	ACCACCACACCTGAAAGCAG	AGCTGGACACTGTCTCAAAG
*Lc3*	NM_022867	GGTCCAGTTGTGCCTTTATTGA	GTGTGTGGGTTGTGTACGTCG
*Sqstm1*	NM_001393885	CTAGGCATCGAGGTTGACATT	CTTGGCTGAGTACCACTCTTATC
*Ulk1*	NM_001108341	GGCTTACAGACTGCCATTGA	GATACCACGCTGGCCTTATAC
*Ppia*	NM_017101	TCAAACACAAATGGTTCCCAGT	ATTCCTGGACCCAAAACGCT

**Table 2:** Lipid parameters in the liver.

Parameter	Group		
	STD-VH	CAF-VH	CAF-GSPE
TAG (mg/g)	3.755 (3.373–3.215)	11.686 (11.335–12.118) *	9.914 (9.633–9.985) &
Cholesterol (mg/g)	1.207 (1.130–1.251)	1.395 (1.314–1762) *	1.390 (1.247–1.481)
Total lipids (mg/g)	63.719 (54.201–72.582)	89.127(77.189–105.920) *	81.662 (75.031–88.872)

Lipid parameters in the livers of Fischer 344 rats fed an STD diet and exposed to three different photoperiods for 9 weeks, supplemented with vehicle or GSPE for the last 4 weeks. Data are given as medians (quartile 1 [Q1]–quartile 3 [Q3]) (*n* = 16), determined using the Mann–Whitney test. * indicates significant differences between STD-VH and CAF-VH. & indicates significant differences between CAF-VH and CAF-GSPE (*p* < 0.05).

**Table 3:** Differential hepatic metabolites in STD-VH, CAF-VH, and CAF-GSPE rats’ VIP scores.

Group	Metabolite	VIP Score
STD-VH	Glutamine	1.98
	Serine	1.90
	Lactic acid	1.82
	Malic acid	1.50
	Glucose-6-phosphate	1.47
	α-Ketoglutaric acid	1.38
	Pyruvic acid	1.32
	Ornithine	1.30
	Aspartic acid	1.23
	Xylonic acid	1.03
CAF-VH	Pyruvic acid	2.43
	Lactic acid	2.01
	Alanine	1.53
	Citric acid	1.45
	3-Hydroxyisobutyric acid	1.40
	α-Ketoglutaric acid	1.37
	Succinic acid	1.13
	Glucose-6-phosphate	1.09
	Malic acid	1.01
CAF -GSPE	Cholesterol	2.11
	Ornithine	1.92
	4-Hydroxyproline	1.63
	Taurine	1.52
	Malic acid	1.41
	Aspartic acid	1.32
	Glycerol-1-phosphate	1.31
	α-Ketoglutaric acid	1.17
	Citric acid	1.13
	Glutamine	1.13
	Glutamic acid	1.11

Differential hepatic metabolites in STD-VH, CAF-VH, and CAF-GSPE groups identified by Partial Least Squares Discriminant Analysis (PLS-DA). Variable importance in projection (VIP) scores indicate the contribution of each metabolite to group discrimination. Only metabolites with VIP > 1.0 are shown.

## Data Availability

The original contributions presented in this study are included in the article/Supplementary Materials. Further inquiries can be directed to the corresponding authors.

## Supplementary Materials

The following supporting information can be downloaded at https://www.mdpi.com/article/10.3390/antiox15060734/s1: Supplementary Note S1: Phenolic profile and quantification of the grape seed proanthocyanidin extract (GSPE) by HPLC-MS/MS. Supplementary Note S2: detailed circadian rhythm analysis for BMAL1 and REV-ERBα. Supplementary Note S3: Detailed Circadian Rhythm Analysis for Autophagy-Related Proteins and Genes. Supplementary Note S4: detailed circadian rhythm analysis for the NRF2/HO-1 antioxidant axis. Supplementary Note S5: detailed circadian rhythm analysis for ER-Stress related genes. Table S1. Main phenolic compounds (flavanols and phenolic acids) of the grape seed proanthocyanidin extract (GSPE) used in this study, analyzed by HPLC-MS/MS. Figure S1. Representative HPLC chromatographic fingerprint of the grape seed proanthocyanidin extract (GSPE; Vitaflavan®-type extract) used throughout the study. Table S2. Circadian parameters and diurnal oscillations estimated from immunoblotted circadian protein in rats. Table S3. Comparison of diurnal oscillations of immunoblotted circadian proteins between STD-VH, CAF-VH and CAF-GSPE groups. Table S4. Circadian parameters and diurnal oscillations estimated from quantified circadian protein in rats. Table S5. Comparison of diurnal oscillations of immunoblotted circadian proteins between STD-VH, CAF-VH and CAF-GSPE groups. Table S6. Circadian metrics and diurnal rhythms inferred from autophagic gene expression in rats. Table S7. Comparison of diurnal oscillations of genes between STD-VH, CAF-VH and CAF-GSPE groups. Table S8. Circadian parameters and diurnal oscillations estimated from quantified circadian protein in rats. Table S9. Comparison of diurnal oscillations from quantified circadian proteins between STD-VH, CAF-VH and CAF-GSPE groups. Table S10. Circadian parameters and diurnal oscillations estimated from endoplasmic reticulum stress related genes in rats. Table S11. Comparison of diurnal oscillations estimated from endoplasmic reticulum stress related genes in rats. Table S12. KEGG pathway enrichment analysis based on VIP scores across the experimental groups (STD-VH, CAF-VH, and CAF+GSPE).

## Abbreviations

The following abbreviations are used in this manuscript:

α-KG	Alpha-ketoglutaric acid
AMPK	AMP-activated protein kinase
AP-1	Activator protein 1
ARE	Antioxidant response element
ARNT	Aryl hydrocarbon receptor nuclear translocator
Atf6	Activating transcription factor 6
BCA	Bicinchoninic acid
BMAL1	Brain and muscle ARNT-like 1
CAF	Cafeteria diet
cDNA	Complementary DNA
Chop	C/EBP homologous protein
CLOCK	Circadian locomotor output cycles kaput
COS	Centre for Omic Sciences
CRY	Cryptochrome
ECL	Enhanced chemiluminescence
ER	Endoplasmic reticulum
FDR	False discovery rate
FOXO	Forkhead box class O
GC-qTOF	Gas chromatography coupled with quadrupole time-of-flight mass spectrometry
Grp78	Glucose-regulated protein 78
GSPE	Grape seed proanthocyanidin extract
HO-1	Heme Oxygenase-1
KEAP1	Kelch-like ECH-associated protein 1
LC3	Microtubule-associated protein 1A/1B-light chain 3
LC3-I	Cytosolic form of LC3
LC3-II	Lipidated form of LC3
MASH	Metabolic dysfunction-associated steatohepatitis
MASLD	Metabolic dysfunction-associated steatotic liver disease
MESOR	Midline Estimating Statistic of Rhythm
mTORC1	Mechanistic target of rapamycin complex 1
NAFLD	Non-alcoholic fatty liver disease
NR1D1	Nuclear receptor subfamily 1 group D member 1
NRF2	Nuclear factor erythroid 2–related factor 2
p-NRF2	Phosphorylated Nuclear Factor Erythroid 2-Related Factor 2
t-NRF2 PCA	Phosphorylated Nuclear Factor Erythroid 2-Related Factor 2 Principal component analysis
PER	Period protein
PERMANOVA	Permutational multivariate analysis of variance
PIC	Protease inhibitor cocktail
PLS-DA	Partial Least Squares Discriminant Analysis
PMSF	Phenylmethanesulfonyl fluoride
Ppia	Peptidylprolyl isomerase A
p-62	Ubiquitin-binding protein p62
PVDF	Polyvinylidene difluoride
qPCR	Quantitative polymerase chain reaction
REV-ERBα	Nuclear receptor subfamily 1 group D member 1
RIPA	Radioimmunoprecipitation assay buffer
ROR	Retinoic acid-related orphan receptor
ROS	Reactive oxygen species.
RT-qPCR	Reverse transcription quantitative polymerase chain reaction
SEM	Standard error of the mean
SDS	Sodium dodecyl sulfate
SQSTM1	Sequestosome 1
STD	Standard diet
TAG	Triacylglycerol
TBST	Tris-buffered saline with Tween 20
TCA cycle	Tricarboxylic acid cycle
UPR	Unfolded protein response
Ulk1	Unc-51 like autophagy activating kinase 1
VH	Vehicle
VIP	Variable importance in projection
ZT	Zeitgeber time
