# Supplementary material for: Grape Seed Proanthocyanidins Enhance Time-Dependent HO-1 Activation and Improve Redox Homeostasis in Obesity-Induced Hepatic Dysfunction
Source: Antioxidants (Basel). 2026 Jun 9;15(6):734. doi: 10.3390/antiox15060734 (PMC13295885; doi:10.3390/antiox15060734)
Supplement: Supplementary file 1 [file antioxidants-15-00734-s001.zip › antioxidants-4281376-supplementary.pdf]

Supplementary data for "Grape seed proanthocyanidins enhance time-dependent HO-1 activation and improve redox homeostasis in obesity-induced hepatic dysfunction"

Supplementary Note S1: Phenolic profile and quantification of the grape seed proanthocyanidin extract (GSPE) by HPLC-MS/MS.

Table S1. Main phenolic compounds (flavanols and phenolic acids) of the grape seed proanthocyanidin extract (GSPE) used in this study, analyzed by HPLC-MS/MS.

Phenolic compound	(M-H)-	Calibration curve	Total amount (mg/g)	SD
**Protocatechuic acid (PCA)**	153.0187	y = 1E+06x	1.40	0.25
**Catechin**	289.0712	y = 1E+06x	51.88	5.56
**Epicatechin**	289.0712	y = 935152x	62.86	8.32
**Gallic acid**	169.0136	y = 287040x	44.66	7.76
**Kaempferol-3-glucoside**	447.0927	y = 1E+06x	0.50	0.02
**Naringenin-7-glucoside**	433.1135	y = 3E+06x	0.64	0.08
**p-Coumaric acid**	163.0395	y = 2E+06x	0.09	0.01
**Quercetin**	301.0348	y = 2E+06x	0.05	0.01
**Quercetin-3-O-galactoside**	463.0877	y = 2E+06x	0.43	0.05
**Vanillic acid**	167.0342	y = 1E+06x	0.09	0.01
**Procyanidin dimer**	577.1346	y = 664077x	76.84	15.76
**Procyanidin trimer**	865.1979	y = 664077x	13.04	0.64
**Procyanidin tetramer**	1153.2613	y = 664077x	5.14	0.28
**Dimer gallate**	729.1455	y = 1E+06x	15.22	2.72
**Epicatechin gallate**	441.0821	y = 1E+06x	14.24	2.76
**Epigallocatechin gallate**	457.077	y = 1E+06x	0.06	0.01

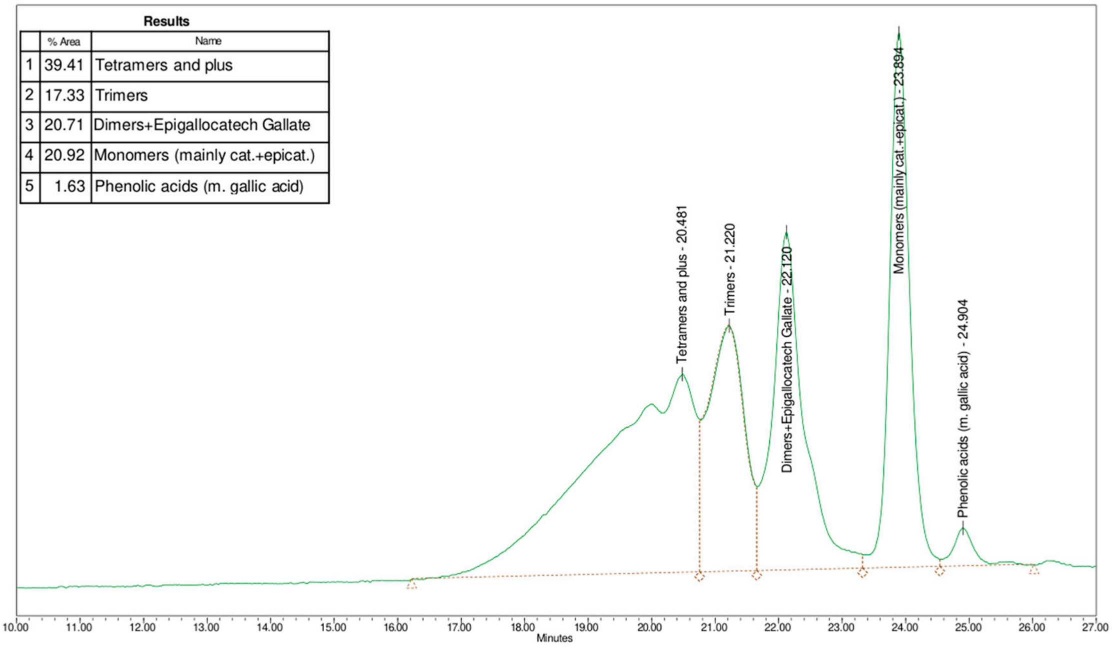

Figure S1. Representative HPLC chromatographic fingerprint of the grape seed proanthocyanidin extract (GSPE; Vitaflavan®-type extract) used throughout the study. The chromatographic profile illustrates the characteristic distribution of flavan-3-ols and oligomeric proanthocyanidins typically found in *Vitis vinifera* seed extracts, including monomeric flavanols (mainly catechin and epicatechin), procyanidin dimers, trimers, tetramers and higher oligomers, together with minor phenolic acids. Peak assignments and relative area percentages are indicated within the chromatogram. The predominance of oligomeric proanthocyanidins is consistent with the targeted HPLC-MS/MS compositional analysis presented in Supplementary Note S1, where the main constituents were identified based on their precursor ions ((M−H)−), calibration curves, and quantitative profiling. This chromatographic fingerprint further supports the compositional consistency, analytical traceability, and reproducibility of the standardized GSPE preparation used in the biological experiments.

Supplementary Note S2: detailed circadian rhythm analysis for BMAL1 and REV-ERBα

This section provides the comprehensive statistical outcomes of the Cosinor-based rhythmometry analysis and CircAnalyst comparisons for the core circadian clock proteins BMAL1 and REV-ERBα. As detailed in **Table S2**, the overall circadian rhythmicity of BMAL1 was not statistically detected in any diet-treatment group (STD-VH: p = 0.299; CAF-VH: p = 0.381; CAF-GSPE: p = 0.179). However, specific rhythmic parameters were identified: the amplitude of BMAL1 oscillation reached significance exclusively in the CAF-GSPE group (p = 0.047), while the acrophase was significant across all groups, occurring at ZT13 in STD-VH rats, ZT9 in CAF-VH rats, and ZT5 in CAF-GSPE rats. Inter-group comparisons (**Table S2**) revealed a statistical tendency toward differences in global rhythmic parameters between the STD-VH and CAF-VH groups (p = 0.067), though no significant recovery was observed when comparing CAF-VH with CAF-GSPE (p = 0.208). Regarding REV-ERBα, intra-group analysis (**Table S1**) confirmed the absence of robust circadian rhythmicity across all experimental groups (p > 0.05). Consequently, subsequent inter-group comparisons (**Table S3**) showed no significant differences in rhythmic parameters for REV-ERBα between STD-VH and CAF-VH, or between CAF-VH and CAF-GSPE groups, indicating this specific marker remained largely unaffected by the interventions.

Table S2. Circadian parameters and diurnal oscillations estimated from immunoblotted circadian protein in rats.

Parameter	Group	Period (h)	*p*	*MESOR*	*Amplitude*	*p (Amplitude)*	*Acrophase (h)*	*p (Acrophase)*
BMAL1	STD-VH	24	0.299	0.328	0.094	0.103	12.825	0.000***
	CAF-VH	24	0.381	0.193	0.063	0.149	9.020	0.001***
	CAF-GSPE	24	0.179	0.250	0.084	0.047	4.741	0.014
REV-ERB	STD-VH	24	0.763	0.931	0.171	0.457	16.181	0.128
	CAF-VH	24	0.822	0.749	0.103	0.528	14.941	0.135
	CAF-GSPE	24	0.957	0.859	0.06#	0.766	2.829	0.826

Acrophase is the time at which the peak of an oscillation occurs ((h): hours); Amplitude represents half the difference between peak and trough values; MESOR is a diurnal rhythm-adjusted mean. The values are the estimation of circadian parameters obtained by cosinor method. *, ** or *** Diurnal oscillations and parameters significantly detected ($p < 0.05$, $p < 0.01$ or $p < 0.001$, respectively). # Indicates tendency ($p < 0.1$).

Table S3. Comparison of diurnal oscillations of immunoblotted circadian proteins between STD-VH, CAF-VH and CAF-GSPE groups

Parameter	Group	*p*	*p*(amplitude)	*p*(acrophase)
BMAL 1	STD-VH vs CAF-VH	0.067#	0.662	0.310
	STD-VH vs CAF+GSPE	0.167	0.883	0.009**
	CAF-VH vs CAF+GSPE	0.208	0.727	0.189
REV-ERB	STD-VH vs CAF-VH	0.866	0.808	0.886
	STD-VH vs CAF+GSPE	0.974	0.715	0.467
	CAF-VH vs CAF+GSPE	0.976	0.868	0.380

The circadian parameter *p*-values are the differences between groups (STD-VH vs CAF-VH, STD-VH Vs CAF+GSPE, CAF-VH Vs CAF+GSPE) for each circadian parameter. The measurements correspond to the inferred circadian metrics obtained through the Cosinor method. ** indicates $p < 0.01$, while # indicates trend or tendency ($0.1 > p > 0.05$). STD-VH, rats fed standard diet and vehicle; CAF-VH, rats fed a cafeteria diet and vehicle; CAF-GSPE, rats fed a cafeteria diet and supplemented with 25 mg/kg b.w. GSPE; h=hours.

Supplementary Note S3: Detailed Circadian Rhythm Analysis for Autophagy-Related Proteins and Genes

This section details the statistical outcomes of the Cosinor-based rhythmometry analysis and pairwise CircAnalyst comparisons for key autophagic markers at both the protein (LC3-II/I ratio, p62, AMPK) and transcriptional (*Lc3*, *Sqstm1*, *Ulk1*) levels. Regarding the protein expression analysis, as shown in **Table S4**, the LC3-II/I ratio displayed a tendency toward overall circadian rhythmicity exclusively in STD-VH rats (p = 0.050), whereas both CAF-fed groups lacked rhythmicity ($p > 0.05$). Significant differences in amplitude and acrophase were observed within the STD-VH group, though inter-group comparisons (**Table S5**) yielded no significant differences in global rhythmic parameters across diet-treatment conditions. For p62, while STD-VH and CAF-VH groups were arrhythmic, GSPE supplementation restored a significant diurnal rhythm in CAF-GSPE livers (p = 0.014), accompanied by a highly significant amplitude (p = 0.0005). Acrophase comparisons revealed a highly significant temporal shift between the CAF-VH and CAF-GSPE groups ($p < 0.001$). AMPK activation profiles lacked circadian rhythmicity across all experimental groups.

Table S4. Circadian parameters and diurnal oscillations estimated from quantified circadian protein in rats

Parameter	Group	Period (h)	*p*	*MESOR*	*Amplitude*	*p (Amplitude)*	*Acrophase (h)*	*p (Acrophase)*
LC3-II/I	STD-VH	24	0.0504#	0.302	0.095	0.0059**	3.553	0.0104*
	CAF-VH	24	0.881	0.246	0.023	0.613	22.107	0.802
	CAF-GSPE	24	0.650	0.291	0.051	0.345	21.901	0.604
p62	STD-VH	24	0.461	0.287	0.038	0.1999	10.375	0.0005***
	CAF-VH	24	0.470	0.273	0.047	0.2058	13.581	0.0006***
	CAF-GSPE	24	0.014*	0.287	0.056	0.0005***	2.126	0.067#
AMPK	STD-VH	24	0.692	5.329	0.997	0.384	2.240	0.610
	CAF-VH	24	0.193	3.440	1.376	0.052	6.799	0.000***
	CAF-GSPE	24	0.529	4.092	1.018	0.247	5.657	0.105

The values are the estimation of circadian parameters obtained by Cosinor method. *, ** or *** Diurnal oscillations and parameters significantly detected ($p < 0.05$, $p < 0.01$ or $p < 0.001$, respectively). # Indicates tendency ($p < 0.1$).

Table S5. Comparison of diurnal oscillations of immunoblotted circadian proteins between STD-VH, CAF-VH and CAF-GSPE groups

Parameter	Group	*p*	*p*(amplitude)	*p*(acrophase)
LC3-II/I	STD-VH vs CAF-VH	0.201	0.203	0.431
	STD-VH vs CAF+GSPE	0.359	0.486	0.143
	CAF-VH vs CAF+GSPE	0.832	0.691	0.982
p62	STD-VH vs CAF-VH	0.642	0.851	0.460
	STD-VH vs CAF+GSPE	0.181	0.537	0.000***
	CAF-VH vs CAF+GSPE	0.270	0.765	0.000***
AMPK	STD-VH vs CAF-VH	0.298	0.784	0.307
	STD-VH vs CAF+GSPE	0.644	0.989	0.547
	CAF-VH vs CAF+GSPE	0.499	0.753	0.762

The circadian parameter *p*-values are the differences between groups (STD-VH vs CAF-VH, STD-VH Vs CAF+GSPE, CAF-VH Vs CAF+GSPE) for each circadian parameter. The measurements correspond to the inferred circadian metrics obtained through the Cosinor method. ** indicates $p < 0.01$, while # indicates trend or tendency ($0.1 > p > 0.05$). STD-VH, rats fed standard diet and vehicle; CAF-VH, rats fed a cafeteria diet and vehicle; CAF-GSPE, rats fed a cafeteria diet and supplemented with 25 mg/kg b.w. GSPE; h=hours.

In reference to gene expression analysis at the transcriptional level (**Table S6**), *Lc3* expression followed a significant circadian rhythm in STD-VH rats ($p = 0.005$) but was arrhythmic in CAF-VH and CAF-GSPE rats. *Lc3* transcript levels peaked at ZT14 in STD-VH and CAF-VH livers, and at ZT13 in CAF-GSPE livers, with significant differences in overall rhythmic parameters detected between STD-VH and CAF-VH groups ($p = 0.010$; **Table S7**). Regarding *Sqstm1* (*p62*), circadian analysis suggested a tendency toward rhythmicity in STD-VH and CAF-GSPE livers ($p = 0.077$

and p = 0.098, respectively), whereas CAF-VH livers remained arrhythmic. *Sqstm1* transcripts peaked at ZT7 in STD-VH livers, shifted to ZT11 in CAF-VH livers, and were seemingly restored to ZT7 with GSPE supplementation, accompanied by significant amplitudes in the STD-VH and CAF-GSPE groups. Finally, while *Ulk1* expression lacked global rhythmicity across all groups, significant acrophases were detected in CAF-VH and CAF-GSPE groups, with a statistical trend indicating a delayed acrophase in CAF-VH versus STD-VH livers (p = 0.050).

Table S6. Circadian metrics and diurnal rhythms inferred from autophagic gene expression in rats

Parameter	Group	Period (h)	*p*	*MESOR*	*Amplitude*	*p (Amplitude)*	*Acrophase (h)*	*p (Acrophase)*
Lc3	STD-VH	24	0.005*	1.399	0.634	0.000***	14.098	0.000***
	CAF-VH	24	0.333	1.210	0.284	0.121	14.420	0.000***
	CAF-GSPE	24	0.109	1.399	0.404	0.022*	13.436	0.000***
Sqstm1	STD-VH	24	0.077#	0.962	0.223	0.012*	6.984	0.000***
	CAF-VH	24	0.566	0.835	0.113	0.276	11.185	0.001***
	CAF-GSPE	24	0.098#	0.908	0.167	0.018*	7.342	0.000***
	STD-VH	24	0.668	0.669	0.125	0.361	5.736	0.171
Ulk 1	CAF-VH	24	0.582	0.655	0.108	0.288	16.759	0.044*
	CAF-GSPE	24	0.174	0.929	0.206	0.045*	18.743	0.006***

The values are the estimation of circadian parameters obtained by cosinor method. *,** or *** Diurnal oscillations and parameters significantly detected ($p < 0.05$, $p < 0.01$ or $p < 0.001$ respectively). # Indicates tendency ($p < 0.1$).

Table S7. Comparison of diurnal oscillations of genes between STD-VH, CAF-VH and CAF-GSPE groups

Parameter	Group	*p*	*p*(amplitude)	*p*(acrophase)
Lc3	STD-VH vs CAF-VH	0,.010*	0.145	0.895
	STD-VH vs CAF+GSPE	0.007**	0.330	0.723
	CAF-VH vs CAF+GSPE	0.170	0.637	0.732
Sqstm1	STD-VH vs CAF-VH	0.172	0.419	0.251
	STD-VH vs CAF+GSPE	0.056	0.624	0.876
	CAF-VH vs CAF+GSPE	0.349	0.663	0.288
Ulk 1	STD-VH vs CAF-VH	0.289	0.921	0.050*
	STD-VH vs CAF+GSPE	0.143	0.634	0.011*
	CAF-VH vs CAF+GSPE	0.415	0.496	0.627

The circadian parameter *p*-values are the differences between groups (STD-VH vs. CAF-VH STD-VH vs. CAF+GSPE, CAF-VH vs. CAF+GSPE) for each circadian parameter. The measurements correspond to the inferred circadian metrics obtained through the Cosinor method. ** indicates $p < 0.01$, while # indicates trend or tendency ($0.1 > p > 0.05$). STD-VH, rats fed standard diet and vehicle; CAF-VH, rats fed a cafeteria diet and vehicle; CAF-GSPE, rats fed a cafeteria diet and supplemented with 25 mg/kg b.w. GSPE; h=hours.

Supplementary Note S4: detailed circadian rhythm analysis for the NRF2/HO-1 antioxidant axis

Following the evaluation of the core clock and autophagic markers, this section details the mathematical estimates (Cosinor method) and inter-group statistical comparisons (CircAnalyst) for the antioxidant axis, specifically focusing on phosphorylated NRF2 (pNRF2) and its

downstream target, heme oxygenase-1 (HO-1). As detailed in **Table S8**, robust circadian rhythmicity for pNRF2 was not statistically detected in any of the experimental groups ($p > 0.05$). Consequently, subsequent inter-group comparisons (**Table S9**) confirmed that the global oscillatory parameters for pNRF2 remained unmodulated by either the obesogenic diet or GSPE supplementation. This indicates that, despite qualitative trends, pNRF2 activation does not follow a mathematically consolidated circadian pattern under these experimental conditions. In contrast, the downstream effector HO-1 exhibited a significant physiological circadian rhythm in healthy STD-VH rats ($p = 0.010$), a temporal organization that was completely lost under the obesogenic CAF diet ($p = 0.371$). While GSPE supplementation did not fully restore a global 24-h rhythm to baseline levels, it induced a highly specific and functional temporal pattern. Specifically, the CAF-GSPE group displayed a significant amplitude ($p = 0.045$) and a highly significant acrophase ($p < 0.001$) peaking closely to ZT13 (**Table S8**). Furthermore, inter-group analysis (**Table S9**) confirmed significant differences in overall rhythmic parameters between the STD-VH and both CAF-fed groups. Crucially, GSPE supplementation induced a significant temporal shift (acrophase) when compared directly to the untreated CAF group ($p = 0.015$). Taken together, this narrative confirms a treatment-specific enhancement of time-dependent antioxidant activation, reinforcing defenses precisely at the onset of the active phase.

Table S8. Circadian parameters and diurnal oscillations estimated from quantified circadian protein in rats

Parameter	Group	Period (h)	*p*	*MESOR*	*Amplitude*	*p (Amplitude)*	*Acrophase (h)*	*p (Acrophase)*
	STD-VH	24	0.758	0.684	0.111	0.452	4.476	0.378
p-NRF2	CAF-VH	24	0.916	0.785	0.112	0.673	14.583	0.299
	CAF-GSPE	24	0.990	0.705	0.025	0.889	3.113	0.909
	STD-VH	24	0,010*	0,666	0,398	0,000***	2,585	0,013**
HO-1	CAF-VH	24	0,371	0,468	0,182	0,144	5,067	0,052
	CAF-GSPE	24	0,173	0,698	0,207	0,045*	12,816	0,000***

The values are the estimation of circadian parameters obtained by cosinor method. *. ** or *** Diurnal oscillations and parameters significantly detected ($p < 0.05$. $p < 0.01$ or $p < 0.001$. respectively). # Indicates tendency ($p < 0.1$).

Table S9. Comparison of diurnal oscillations from quantified circadian proteins between STD-VH, CAF-VH and CAF-GSPE groups

Parameter	Group	*p*	*p*(amplitude)	*p*(acrophase)
p-NRF2	STD-VH vs CAF-VH	0.978	0.998	0.332
	STD-VH vs CAF+GSPE	0.991	0.708	0.958
	CAF-VH vs CAF+GSPE	0.995	0.785	0.747
HO-1	STD-VH vs CAF-VH	0.018*	0.193	0.356
	STD-VH vs CAF+GSPE	0.013*	0.203	0.000**
	CAF-VH vs CAF+GSPE	0.117	0.880	0.015

The circadian parameter *p*-values are the differences between groups (STD-VH vs CAF-VH, STD-VH Vs CAF+GSPE, CAF-VH Vs CAF+GSPE) for each circadian parameter. The measurements correspond to the inferred circadian metrics obtained through the Cosinor method. ** indicates $p < 0.01$, while # indicates trend or tendency ($0.1 > p > 0.05$). STD-VH, rats fed standard diet and vehicle; CAF-VH, rats fed a cafeteria diet and vehicle; CAF-GSPE, rats fed a cafeteria diet and supplemented with 25 mg/kg b.w. GSPE; h=hours.

Supplementary Note S5: detailed circadian rhythm analysis for ER-Stress related genes

Continuing the molecular characterization of the hepatic stress response, this section details the mathematical estimates (Cosinor method) and inter-group statistical comparisons (CircAnalyst) for key Endoplasmic Reticulum (ER) stress-related genes: *Grp78*, *Atf6*, and *Chop*. As detailed in **Table S10**, physiological 24-h circadian rhythmicity for these ER-stress markers was generally weak or absent in both the healthy (STD-VH) and obesogenic (CAF-VH) control groups. However, specific gene-dependent oscillatory features were observed, and GSPE supplementation exerted a modest modulatory effect on their temporal profiles. For instance, while neither STD-VH nor CAF-VH groups exhibited global 24-h rhythmicity for *Atf6*, GSPE supplementation successfully induced a statistically significant circadian rhythm in CAF-GSPE livers ($p = 0.049$). This temporal organization was accompanied by a significant amplitude ($p = 0.006$) and a robust acrophase ($p < 0.001$) peaking at ZT16. Similarly, regarding *Grp78*, a marginal qualitative increase in absolute transcript levels was noted in CAF-VH livers at ZT7 compared to STD-VH ($p = 0.0571$). In terms of temporal oscillation, circadian analysis indicated a trend toward global rhythmicity exclusively in the CAF-GSPE group (*p* = 0.081$), which also presented significant amplitude ($p = 0.013$) and acrophase ($p < 0.001$). Peak *Grp78* expression occurred at ZT10 in STD-VH, ZT9 in CAF-VH, and ZT8 in CAF-GSPE. Finally, *Chop* global circadian rhythmicity was absent across all groups. Nevertheless, significant amplitudes were detected in CAF-VH ($p = 0.046$), and significant acrophases were observed in both CAF-VH ($p < 0.001$) and CAF-GSPE ($p = 0.016$) groups, with peaks occurring later in the day (ZT16–ZT17) compared to the standard diet (ZT9). When analyzing inter-group variations (**Table S11**), no significant differences were found in the overall rhythmic parameters of *Grp78* and *Atf6* across diet or treatment conditions. For *Chop*, while amplitude and acrophase were not individually distinct between groups, CircAnalyst revealed a significant difference in the global rhythmic parameters between the CAF-VH and CAF-GSPE groups ($p = 0.043$).

Table S10. Circadian parameters and diurnal oscillations estimated from endoplasmic reticulum stress related genes in rats

Parameter	Group	Period (h)	*p*	*MESOR*	*Amplitude*	*p (Amplitude)*	*Acrophase (h)*	*p (Acrophase)*
Grp78	STD-VH	24	0.973	1.068	0.030	0.816	9.730	0.554
	CAF-VH	24	0.492	1.453	0.273	0.221	8.822	0.005**
	CAF-GSPE	24	0.081#	1.231	0.306	0.013*	7.558	0.000***
Atf6	STD-VH	24	0.184	1.132	0.278	0.049*	12.541	0.000***
	CAF-VH	24	0.184	1.353	0.434	0.128	15.440	0.001***
	CAF-GSPE	24	0.049*	1.127	0.255	0.006*	15.776	0.000***
Chop	STD-VH	24	0.786	1.041	0.113	0.484	8.877	0.104
	CAF-VH	24	0.176	1.281	0.403	0.046*	15.858	0.000***
	CAF-GSPE	24	0.468	0.865	0.138	0.205	16.766	0.016

Acrophase is the time at which the peak of an oscillation occurs ((h: hours); Amplitude is the difference between the peak and the mean value of a wave; MESOR is a diurnal rhythm-adjusted mean. The values are the estimation of circadian parameters obtained by cosinor method. *, ** or *** Diurnal oscillations and parameters significantly detected ($p < 0.05$, $p < 0.01$ or $p < 0.001$, respectively). # Indicates tendency ($p < 0.1$).

Table S11. Comparison of diurnal oscillations estimated from endoplasmic reticulum stress related genes in rats

Parameter	Group	*p*	*p (Amplitude)*	*p (Acrophase)*
Grp78	STD-VH vs CAF-VH	0.273	0.344	0.969
	STD-VH vs CAF+GSPE	0.216	0.121	0.893
	CAF-VH vs CAF+GSPE	0.278	0.897	0.708
Atf6	STD-VH vs CAF-VH	0.318	0.623	0.431
	STD-VH vs CAF+GSPE	0.111	0.895	0.182
	CAF-VH vs CAF+GSPE	0.273	0.551	0.927
Chop	STD-VH vs CAF-VH	0.262	0.263	0.276
	STD-VH vs CAF+GSPE	0.656	0.900	0.190
	CAF-VH vs CAF+GSPE	0.043*	0.248	0.848

Acrophase is the time at which the peak of an oscillation occurs; Amplitude is the difference between the peak and the mean value of a wave. The circadian parameter *p*-values are the differences between groups (STD-VH vs CAF-VH, STD-VH vs CAF+GSPE, CAF-VH vs CAF+GSPE) for each circadian parameter. The measurements correspond to the inferred circadian metrics obtained through the Cosinor method. ** indicates $p < 0.01$, while # indicates trend or tendency ($0.1 > p > 0.05$). STD-VH, rats fed standard diet and vehicle; CAF-VH, rats fed a cafeteria diet and vehicle; CAF-GSPE, rats fed a cafeteria diet and supplemented with 25 mg/kg b.w. GSPE; h=hours.

Table S12. KEGG pathway enrichment analysis based on VIP scores across the experimental groups (STD-VH, CAF-VH, and CAF+GSPE**)**

Group	Metabolite	Total	Hits	FDR
**STD-VH**	Arginine biosynthesis	14	4	4.5e-05
	Alanine, aspartate and glutamate metabolism	28	4	0.000444
	Glyoxylate and dicarboxylate metabolism	32	4	0.000514
	D-Amino acid metabolism	14	3	0.00103
	Citrate cycle (TCA cycle)	20	3	0.00253
	Pyruvate metabolism	22	3	0.00283
	Glycolysis / Gluconeogenesis	24	3	0.00317
	Lipoic acid metabolism	28	2	0.106
	Neomycin, kanamycin and gentamicin2		1	0.106
	biosynthesis	32	2	0.11
	Glycine, serine and threonine metabolism	33	2	0.11
	Cysteine and methionine metabolism	35	2	0.11
	Arginine and proline metabolism			
**CAF-VH**	Citrate cycle (TCA cycle)	20	5	2.29e-06
	Alanine, aspartate and glutamate metabolism	28	5	7.12e-06
	Pyruvate metabolism	22	3	0.00555
	Glycolysis / Gluconeogenesis	24	3	0.00555
	Glyoxylate and dicarboxylate metabolism	32	3	0.0106
	Butanoate metabolism	15	2	0.0425

**CAF-GSPE**	Arginine biosynthesis	14	5	5.95e-07
	Alanine, aspartate and glutamate metabolism	28	5	1.41e-05
	Glyoxylate and dicarboxylate metabolism	32	4	0.000844
	D-Amino acid metabolism	14	3	0.00146
	Citrate cycle (TCA cycle)	20	3	0.00358
	Nitrogen metabolism	6	2	0.00779
	Arginine and proline metabolism	35	3	0.0139
	Butanoate metabolism	15	2	0.0396
	Histidine metabolism	16	2	0.0401

Notes: FDR (False Discovery Rate) indicates the adjusted p-value using the Benjamini-Hochberg method. Bold values indicate significant enrichment (FDR < 0.05). Total and Hits represent the number of metabolites in the KEGG pathway and those identified in the study, respectively
